# Soybean pod shattering resistance allele pdh1 and marker‐assisted selection

**DOI:** 10.1002/pei3.70003

**Published:** 2024-08-12

**Authors:** Dora Shimbwambwa, Christabel Nachilima, Swivia Hamabwe, Kuwabo Kuwabo, Godfree Chigeza, Kristin Bilyeu, Kelvin Kamfwa

**Affiliations:** ^1^ Department of Plant Sciences University of Zambia Lusaka Zambia; ^2^ International Institute of Tropical Agriculture (IITA) Lusaka Zambia; ^3^ Plant Genetics Research Unit United States Department of Agriculture (USDA)−Agricultural Research Service (ARS) Columbia Missouri USA

**Keywords:** allele, heritability, marker‐assisted selection, pod shattering, resistance

## Abstract

Pod shattering is a major production constraint of soybean [*Glycine max* (L.)]. The objectives of this study were to (i) estimate heritability for pod shattering resistance, (ii) determine the frequency of the pod shattering resistance allele *pdh1* in the International Institute for Tropical Agriculture (IITA) soybean germplasm and Zambian commercial varieties, and (iii) determine the effectiveness of the DNA marker for the pod shattering resistance allele *pdh1*. A total of 59 genotypes were evaluated for pod shattering in field trials conducted in Malawi and Zambia and genotyped with a marker for *pdh1*. TGx2002‐8FM and TGx2002‐9FM were the most resistant among genotypes in early and medium maturity classes and can be used for genetic enhancement of pod shattering resistance in these specific maturity classes. Narrow sense heritability estimates for pod shattering ranged from 0.27 to 0.80. Of the 59 genotypes, 57 (96.6%) carried the resistance allele *pdh1* while only two genotypes (3.6%) carried the susceptible allele, suggesting near‐fixation of the resistance allele *pdh1* in the IITA germplasm. The marker for *pdh1* was highly effective in selecting resistant genotypes.

## 1 INTRODUCTION

Soybean [*Glycine max* (L.) Merr.] is a major source of protein and edible oil globally. Pod dehiscence, also known as shattering or splitting of pods, when they have dried, is a seed dispersal mechanism that is useful for self‐propagation of the wild soybean species but is undesirable in domesticated soybean. Yield losses due to pod shattering in soybean may range from 34% to 99% depending on how long harvesting is delayed after maturity, the environmental conditions during harvest and the genotype (Krisnawati & Adie, 2017; Tukamuhabwa et al., 2000). During domestication and breeding, there has been strong selection against pod shattering to control yield losses.

Development and use of pod shattering‐resistant varieties is the most cost‐effective control strategy, and genetic variation for pod shattering resistance exists within cultivated soybean to support development of resistant varieties (Miranda et al., 2019). Both genetic and environmental conditions control pod shattering in soybean (Miranda et al., 2019). Environmental factors such as high ambient temperature and low humidity induce soybean pod shattering (Bara et al., 2013; Caviness, 1965). Varietal differences in pod dehiscence have been attributed partly to differences in the tension of cells of the inner sclenchyma layer as pods begin to dry (Tiwari & Bhatia, 1995; Zhang et al., 2018). The genetic basis of this resistance has been widely studied and genomic regions controlling pod‐shattering resistance have been identified (Bailey et al., 1997; Dong et al., 2014; Funatsuki et al., 2014; Yamada et al., 2009). A major gene (*Pdh1*) controlling pod shattering has been identified on chromosome 16 and has been cloned (Funatsuki et al., 2008, 2014). The functional allele (*Pdh1*) of this gene is associated with pod shattering susceptibility, whereas the nonfunctional allele (*pdh1*) is associated with shattering resistance. *Pdh1* encodes a dirigent‐like protein, which is highly expressed in the lignin‐rich inner sclerenchyma of pod wall where it plays a significant role in the lignification of the pod wall (Funatsuki et al., 2014). Pod walls with high levels of lignin are predisposed to increased torsion, which leads to shattering of dry pods under low humidity (Zhang et al., 2018). A premature stop codon in the *Pdh1* gene causes a loss of its lignin development function resulting in the *pdh1* variant allele conditioning resistance to pod shattering. *Phd1* is a major‐effect gene for this quantitative trait that controls about 42% of variation in pod shattering resistance (Bailey et al., 1997). Fine mapping studies have narrowed genomic regions for these QTL and enabled identification of candidate genes. For example, Gao and Zhu (2013) narrowed down the qPDH1 QTL for pod shattering resistance to 47 kbp region and identified the gene Glyma16g25600, which encodes the bZIP‐type transcription factor as the candidate gene. Funatsuki et al. (2014) identified the gene Glyma16g25580 encoding a dirigent‐like protein as a candidate gene associated with the large effect QTL pdh1 for resistance to pod shattering in soybeans.

The frequency of *pdh1* has previously been studied in a variety of genetic backgrounds, and the results of these studies have been mixed. For example, *pdh1* was highly frequent (>80%) among both old and modern North American germplasm, but less frequent (<20%) in the old and modern Japanese germplasm (Funatsuki et al., 2014). The International Institute for Tropical Agriculture (IITA) is a CGIAR center, which has a mandate for the genetic improvement of soybean in Africa and other tropical environments (Chigeza et al., 2019). Since its inception in 1974, the IITA soybean breeding program has been breeding for pod shattering resistance based on phenotypic selection (Chigeza et al., 2019). A survey of released soybean varieties in Ghana demonstrated two of the seven had shatter‐susceptible *Pdh1* alleles, and a broader survey of older IITA germplasm had 22.5% with *Pdh1* alleles (Miranda et al., 2019). The frequency of *pdh1* in recent IITA soybean germplasm and some of the commercial varieties in Zambia is not known. Knowledge of the frequency of *pdh1* in IITA germplasm could enhance breeding efforts for pod shattering resistance in Africa. DNA markers for *Pdh1*/*pdh1* have been developed and recommended for use in marker‐assisted selection for pod‐shattering resistance (Miranda et al., 2019). Effectiveness of this marker in selection may be affected by the quantitative nature of the trait. Therefore, before deployment of marker‐assisted selection (MAS), validating the effectiveness of this marker for control of pod shatter susceptibility in our environments is important. The objectives of this study were to (i) estimate heritability for pod shattering resistance, (ii) determine the frequency of the pod shattering resistance allele pdh1 in the IITA soybean germplasm and Zambian commercial varieties, and (iii) determine the effectiveness of the DNA marker for the pod shattering resistance allele *Pdh1* to select for pod shattering resistance.

## 2 MATERIALS AND METHODS

### 2.1 Plant material

A total of 59 soybean genotypes were used in the current study. Of this number, 55 were sourced from IITA and were fixed breeding lines that were developed using the single‐seed descent method. The other four (Safari, Lukanga, Kafue, and Dina) were commercial varieties sourced from private and public breeding programs in Zambia (Table 1). The private seed companies SEEDCO and Synergy own the commercial varieties Safari and Dina, respectively. Kafue is a public variety that was developed by IITA, whereas Lukanga is also a public variety owned by Zambia Agricultural Research Institute (ZARI). The four commercial varieties are widely grown in Zambia.

**TABLE 1:** The 59 soybean genotypes were evaluated for pod shattering in field trials and genotyped for the *Pdh1* DNA marker for pod shattering resistance. The 59 genotypes were categorized into IITA early maturing breeding lines and four commercial varieties (T1), IITA medium maturing breeding lines and four commercial varieties (T2), and IITA late‐maturing breeding lines and four commercial varieties (T3) soybean genotypes.

Genotype	Shattering score	Marker	Source
*T1*			
Dina	1.0	R	Synegy
Kafue	2.3	R	IITA
Lukanga	2.3	R	ZARI
SC Safari	1.3	R	SEEDCO
TGx2001‐16DM	1.0	R	IITA
TGx2001‐18FM	3.0	R	IITA
TGx2001‐20 M	2.3	R	IITA
TGx2001‐22DM	2.0	R	IITA
TGx2002‐14DM	1.7	R	IITA
TGx2002‐3DM	1.7	R	IITA
TGx2002‐6FM	3.7	S	IITA
TGx2002‐8FM	1.0	R	IITA
TGx2014‐15FM	3.0	R	IITA
TGx2014‐24FM	2.7	R	IITA
TGx2014‐31FM	2.3	R	IITA
TGx2014‐33FM	3.3	R	IITA
TGx2014‐34FM	3.0	R	IITA
TGx2014‐38FM	2.0	R	IITA
TGx2014‐9FM	2.0	R	IITA
*T2*			
Kafue	1.8	R	IITA
Lukanga	1.9	R	ZARI
Dina	1.3	R	Synergy
SC Safari	1.8	R	SEEDCO
TGx1987‐62F	1.9	R	IITA
TGx2001‐15DM	1.7	R	IITA
TGx2001‐18DM	1.9	R	IITA
TGx2001‐1DM	1.6	R	IITA
TGx2001‐24DM	1.4	R	IITA
TGx2001‐26FM	1.6	R	IITA
TGx2001‐2DM	1.8	R	IITA
TGx2001‐6FM	1.8	R	IITA
TGx2001‐8DM	1.8	R	IITA
TGx2001‐9DM	1.7	R	IITA
TGx2002‐17DM	2.3	R	IITA
TGx2002‐23DM	1.9	R	IITA
TGx2002‐4DM	1.8	R	IITA
TGx2002‐5FM	2.0	R	IITA
TGx2002‐7FM	2.0	R	IITA
TGx2002‐9FM	1.3	R	IITA
TGx2014‐16FM	1.8	R	IITA
TGx2014‐17FM	1.7	R	IITA
TGx2014‐19FM	2.3	R	IITA
TGx2014‐5GM	1.8	R	IITA
*T3*			
Dina	1.2	R	Synergy
Kafue	1.4	R	IITA
Lukanga	1.7	R	ZARI
SC Safari	1.5	R	SEEDCO
TGx2001‐10DM	1.5	R	IITA
TGx2001‐11DM	1.6	R	IITA
TGx2001‐13DM	1.8	R	IITA
TGx2001‐13FM	1.6	R	IITA
TGx2001‐14DM	1.6	R	IITA
TGx2001‐14FM	1.7	R	IITA
TGx2001‐19DM	1.3	R	IITA
TGx2001‐24FM	1.4	R	IITA
TGx2001‐5DM	1.5	R	IITA
TGx2001‐5FM	1.5	R	IITA
TGx2001‐8FM	1.9	R	IITA
TGx2002‐35FM	1.4	R	IITA
TGx2002‐3FM	2.8	S	IITA
TGx2002‐6DM	1.9	R	IITA
TGx2014‐21FM	1.5	R	IITA
TGx2014‐23FM	1.3	R	IITA
TGx2014‐27FM	1.9	R	IITA
TGx2014‐43FM	1.5	R	IITA
TGx2014‐44FM	1.4	R	IITA
TGx2014‐4FM	1.6	R	IITA

Abbreviations: IITA, International Institute for Tropical Agriculture; R, pod shattering resistance allele (*pdh1*); S, pod shattering susceptible allele (*Pdh1*); ZARI, Zambia Agricultural Research Institute.

### 2.2 Field trials

The 55 IITA breeding lines genotypes were categorized into three maturity groups T1 (85–99 days), T2 (100–111 days), and T3 (112–120 days). The number of genotypes in T1 was 19 (15 IITA breeding lines and four commercial varieties), T2 had 24 (20 IITA breeding lines and four commercial checks), whereas T3 had 24 (20 IITA breeding lines and four commercial checks) (Table 1). The 59 genotypes were evaluated for pod shattering resistance in field trials conducted in Malawi and Zambia. Field trials were conducted in two locations (Bvumbwe and Chitedze) in Malawi and two locations (Kabwe and SARAH‐IITA) in Zambia. T1, T2, and T3 genotypes were planted in separate trials (hereafter referred as T1, T2, and T3 trials) but in the same growing season in all four locations using alpha lattice design with three replications at each location.

Each genotype was planted in a plot comprised of four rows, and each row was 4 m long. The interrow spacing was 60 cm, whereas the intrarow spacing was 5 cm. A compound fertilizer was applied at planting (to supply 25 kg N/ha, 30 kg K_2_O/ha, and 60 kg P_2_O_5_/ha). Preemergence (Metolachlor and Imazethapyr), postemergence (Quizalofop‐p‐ethyl and Fomesafen) herbicides, and mechanical weeding were used to control weeds.

Pod shattering was assessed in the field 2 weeks after physiological maturity using a scoring scale of 1–5 (Krisnawati & Adie, 2017). The score was based on the proportion of shattered pods out of the total pods in a plot. Where 1 = no pod shattering (very resistant), 2 = <25% pod shattering (resistant), 3 = 25%–50% pod shattering (moderately resistant), 4 = 51%–75% pod shattering (highly susceptible), and 5 = >75% pod shattering (very highly susceptible).

### 2.3 Phenotypic data analysis

To assess the significance of genotypic, location, and interaction effects on pod shattering, analysis of variance (ANOVA) was conducted for T1, T2, and T3 trials using PROC MIXED in SAS 9.3 (SAS Institute, 2011) using the following the mixed model:

y=μ+G+L+G*L+R+E,

where *y* is pod shattering, μ is the population mean; *G* is the fixed variable effect of the genotype; *L* is the fixed effect of the location; G*L is the genotype by location interaction effect; *R* is the random effect of a block within a location; and *E* is the residual (error), which is assumed to be normally distributed with mean = 0.

### 2.4 Heritability estimates

The variance component estimates from the ANOVA table were used to estimate broad‐sense heritability for pod shattering in T1, T2, and T3 trials using the following equation:

$$h^{2}=\frac{\sigma_{g}^{2}}{\sigma_{g}^{2}+\sigma_{\mathrm{gl}}^{2}/l+\sigma_{e}^{2}/\mathrm{rl}},$$

where *h* ^2^ is broad‐sense estimate heritability for pod shattering for either T1, T2, or T3; $\sigma_{g}^{2}$ is the genotype variance component, $\sigma_{\mathrm{gl}}^{2}$ is the variance component for genotype × location interaction; $\sigma_{e}^{2}$ is the error variance component; *l* is the number of locations; and *r* is the number of replications within a location.

### 2.5 Genotyping and genotypic data analysis

For genotyping of *Pdh1*, a PCR‐based molecular marker assay was developed by using primers for amplification of the region surrounding the causative single nucleotide polymorphism (SNP) and a Simple Probe that was designed for the T/A SNP (Gm16: 29,601,807 Wm82.a1. and v1/Gm16: 29,944,393 Wm82). The genotyping was conducted as previously described for the *Pdh1* SimpleProbe assay (Miranda et al., 2019).

The average pod shattering value of each genotype from the four locations was used in determining the effectiveness of MAS for pod shattering resistance. A paired *t*‐test was conducted to determine the statistical significance of pod shattering differences between genotypes possessing the resistance allele and susceptible allele.

## 3 RESULTS

**FIGURE 1:**
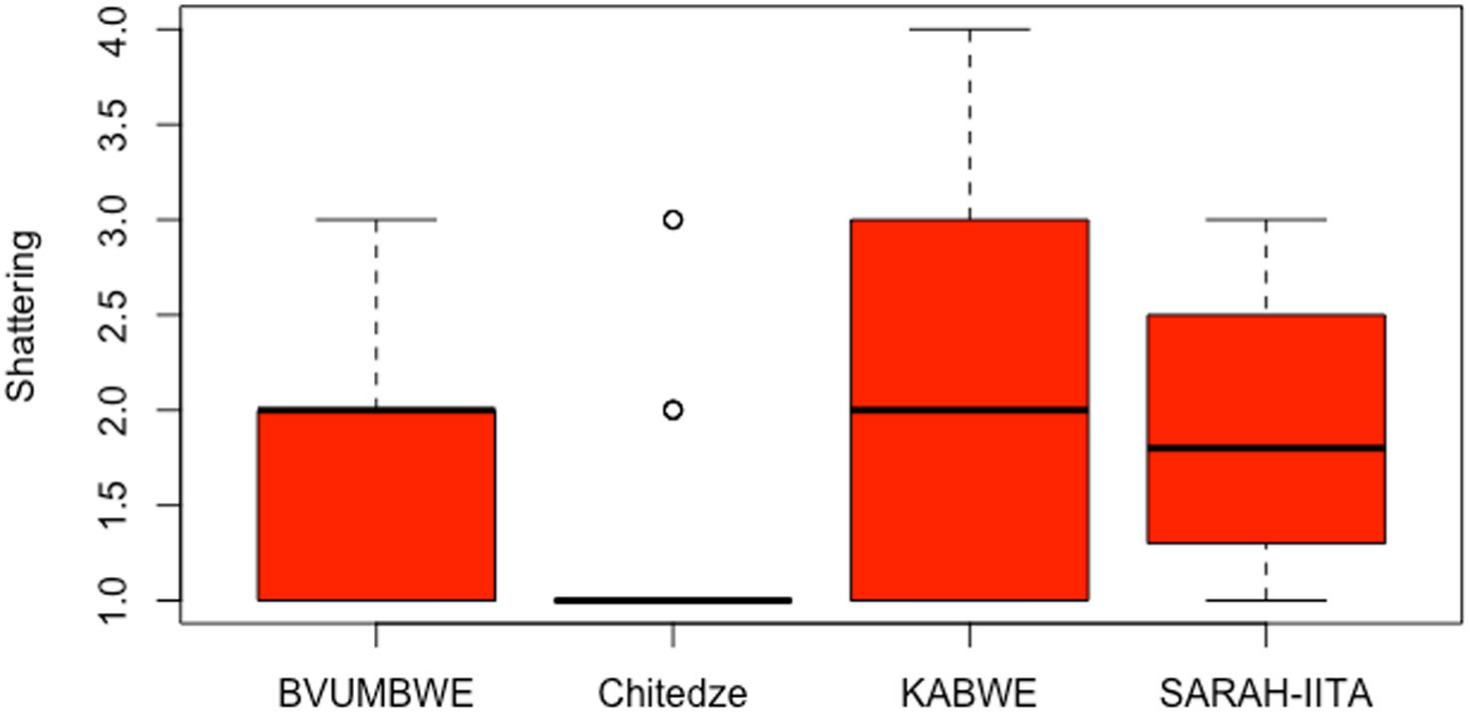
Average pod shattering scores of commercial varieties and 15 early maturing IITA soybean breeding lines (T1) evaluated for pod shattering in four locations in Malawi (Bvumbwe and Chitedze) and Zambia (Kabwe and SARAH‐IITA).

**FIGURE 2:**
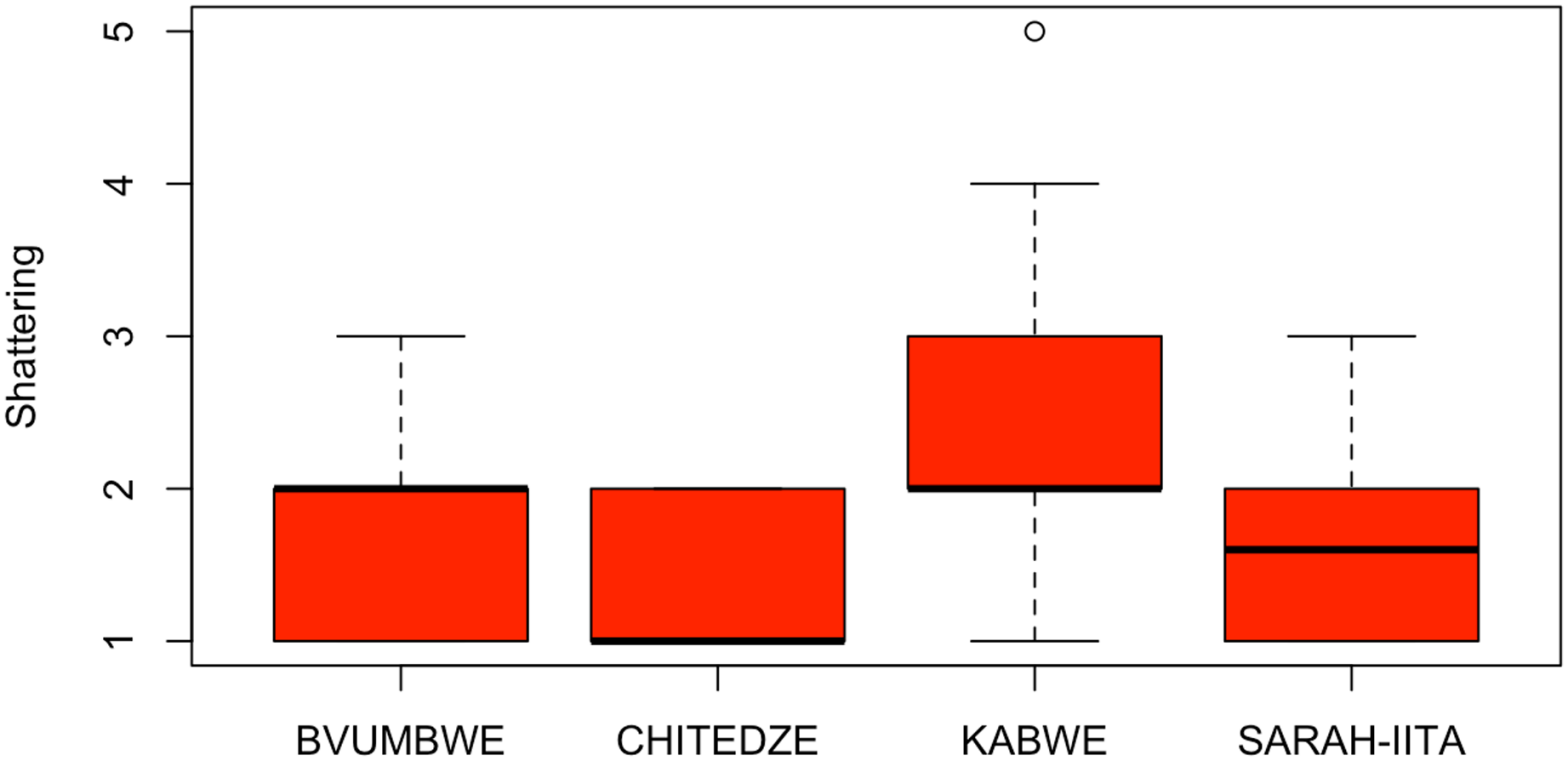
Average pod shattering scores of commercial varieties and 20 medium‐maturing IITA soybean breeding lines (T2) evaluated for pod shattering in four locations in Malawi (Bvumbwe and Chitedze) and Zambia (Kabwe and SARAH‐IITA).

**FIGURE 3:**
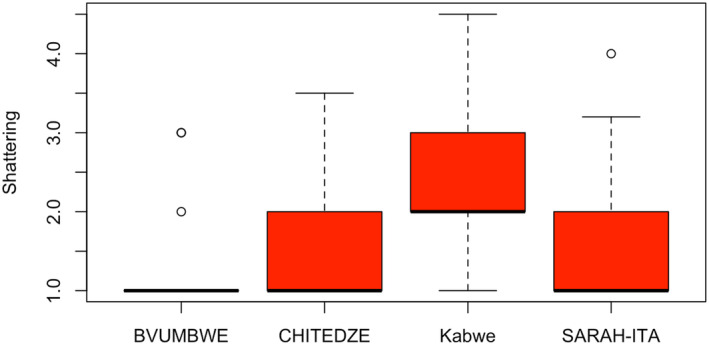
Average pod shattering scores of commercial varieties and 20 late‐maturing IITA soybean breeding lines (T3) evaluated for pod shattering in four locations in Malawi (Bvumbwe and Chitedze) and Zambia (Kabwe and SARAH‐IITA).

**FIGURE 4:**
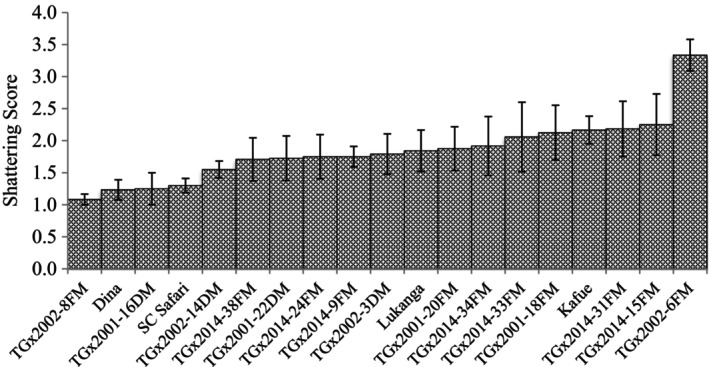
Pod shattering scores of commercial varieties and 15 early maturing IITA soybean breeding lines (T1) evaluated for pod shattering in four locations in Malawi (Bvumbwe and Chitedze) and Zambia (Kabwe and SARAH‐IITA).

Significant (*p* < .05) differences among genotypes in pod shattering were observed in T1 trial (Table 2). Also, the location and genotype × environment (GE) interaction effects were significant (*p* < .05) (Table 2). In Malawi, shattering resistance scores ranged from 1.3 (TGx2001‐22DM) to 2.8 (TGx2002‐6FM), with an average of 1.8 for Bvumbwe, and 1.0–3.0 (TGx2002‐6FM) with a mean of 1.2 for Chitedze. In Zambia, shattering resistance values ranged from 1.0 (TGx2001‐16DM; TGx2002‐8FM) to 3.6 (TGx2002‐6FM), with an average value of 2.2 for Kabwe, and 1 (TGx2001‐16DM; TGx2002‐8FM) to 3.8 (TGx2002‐6FM) with a mean of 2.2 for SARA‐IITA, Figures 1, 2, and 3 show average shattering scores per location for T1, T2, and T3, respectively. The scores for the shattering resistant check (Dina) were 1.6, 1.0, 1.0, and 1.3 for Bvumbwe, Chitedze, Kabwe, and SARA‐IITA, respectively. The T1 average pod shattering score was 1.8. The breeding line TGx2002‐8FM had the lowest average score (1.1) across the four locations, which was lower than that of Dina (1.3) (Figure 4).

**TABLE 2:** Analysis of variance for early maturing (T1), medium maturing (T2), and late maturing (T3) soybean genotypes evaluated for pod shattering in field trials conducted in four locations in Malawi and Zambia in the 2019 growing season.

Source variation	Degrees of freedom	Mean square
*T1 trial*		
Genotype	14	2.981***
Location	3	13.840***
Block	2	0.166^ns^
Genotype: Location	42	0.585***
*T2 trial*		
Genotype	19	0.749***
Location	3	8.355***
Block	2	0.894^ns^
Genotype:Location	57	0.546***
T3 trial		
Genotype	19	1.228***
Location	3	17.408***
Block	2	0.158^ns^
Genotype:Location	57	0.626***

*Note*: T1 trial: early‐maturing soybean varieties and checks. T2 trial: medium‐maturing soybean varieties and checks. T3 trial: late‐maturing soybean varieties and checks. Statistical significance level: ***Significant at *p* < .001; ns, nonsignificant.

**FIGURE 5:**
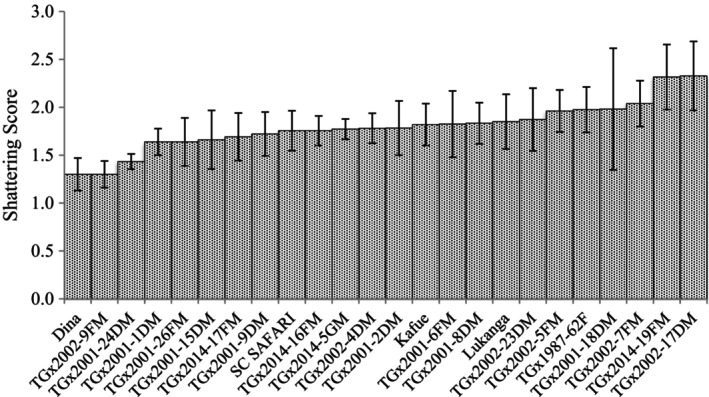
Pod shattering scores of four commercial varieties and 20 medium maturing IITA soybean breeding lines (T2) evaluated for pod shattering in four locations in Malawi (Bvumbwe and Chitedze) and Zambia (Kabwe and SARAH‐IITA).

In T2 trial, there were significant (*p* < .05) differences among genotypes in pod shattering (Table 2). Also, the location and GE effects were significant (*p* < .05). In Malawi, shattering resistance values ranged from 1.0 (TGx2001‐18DM) to 2.3 (TGx2002‐7FM), with an average value of 1.8 for Bvumbwe, and 1.0 (TGx2002‐9FM) to 2.0 (TGx2014‐19FM) with a mean of 1.4 for Chitedze. In Zambia, shattering resistance values ranged from 1.3 (TGx2002‐9FM; TGx2001‐26FM) to 3.6 (TGx2001‐18DM), with an average value of 2.2 for Kabwe, and 1.2 (TGx2002‐9FM; TGx2001‐26FM) to 2.3 (TGx2002‐17DM) with a mean of 1.8 for SARA‐IITA. The scores for the shattering resistant check (Dina) were 1.7, 1.0, 1.0, and 1.5 for Bvumbwe, Chitedze, Kabwe, and SARA‐IITA, respectively. The average shattering score for T2 was 1.8, and the breeding line TGx2002‐9FM had the lowest average shattering score (1.3), which was similar to that of Dina (1.3) (Figure 5).

**FIGURE 6:**
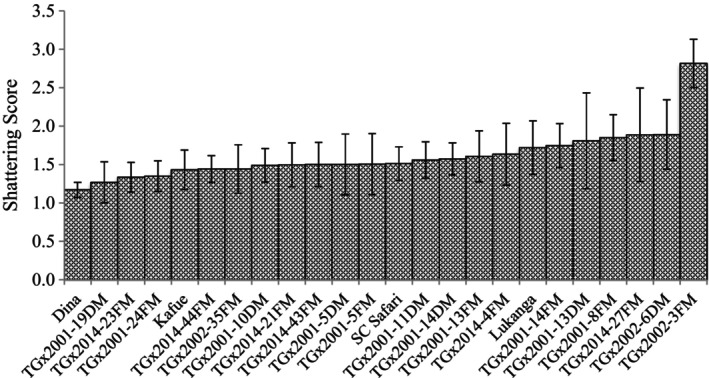
Pod shattering scores of four commercial varieties and 20 late‐maturing IITA soybean breeding lines (T3) evaluated for pod shattering in four locations in Malawi (Bvumbwe and Chitedze) and Zambia (Kabwe and SARAH‐IITA).

In T3 trial, there were significant (*p* < .05) differences among genotypes in pod shattering (Table 2). Also, the location and GE effects were significant (*p* < .05). In Malawi, shattering resistance values ranged from 1.0 to 2.7 (TGx2002‐3FM), with a mean of 1.1 for Bvumbwe, and 1.0 to 3.1 (TGx2002‐3FM) with a mean of 1.5 for Chitedze. In Zambia, shattering resistance values ranged from 1.0 (TGx2001‐19DM) to 3.7 (TGx2014‐27FM), with a mean of 2.3 for Kabwe, and 1.0 (TGx2001‐5DM; TGx2014‐23FM) to 2.4 (TGx2001‐8FM) with a mean of 1.6 for SARA‐IITA. The scores for the shattering resistant check (Dina) were 1.0, 1.3, 1.0, and 1.3 for Bvumbwe, Chitedze, Kabwe, and SARAH‐IITA, respectively. The breeding line TGx2001‐19DM had the lowest average score (1.2) across the four locations (Figure 6).

Broad sense heritability estimates for pod shattering for T1, T2, and T3 trials were 80%, 27%, and 49%, respectively.

Of the 59 IITA soybean lines successfully assayed with the *Pdh1* marker, 57 lines (96.6%) carried the shattering resistant allele while two genotypes (3.4%) carried the shattering susceptible *pdh1* allele. The two genotypes that carried the susceptible allele were TGX 2002‐6FM and TGX 2002‐3FM (Table 1).

The genotypes carrying the shattering susceptible allele had a mean shattering score of 3.1, while genotypes carrying resistant allele had a mean shattering score of 1.7 (Table 3). The *t*‐test results revealed significant (*p* < .001) differences between mean shattering scores of genotypes that carried resistant allele and those that carried the shattering susceptible allele.

**TABLE 3:** Mean scores of pod shattering for genotypes carrying pod shattering resistance allele (*pdh1*) and those carrying the pod shattering susceptible allele (*Pdh1*).

Pod shattering genotypic status	Pod shattering score
Shattering resistance (pdh1)	1.6 ± 0.03
Shattering susceptible (Pdh1)	3.1 ± 0.04
Difference in means	1.5
% Difference	61.3%
*t*‐test	***

*** Significant at *p* < .001.

## 4 DISCUSSION

Pod shattering is a major production constraint of soybean globally and its phenotyping can be challenging. Development of resistant varieties is the most cost‐effective management strategy for pod shattering. This study investigated the frequency of the pod shattering resistance allelic variant allele *pdh1* in IITA germplasm and Zambian commercial varieties, and effectiveness of its marker in MAS for pod shattering.

There were significant differences among genotypes in pod shattering in all three trials, confirming the adequacy of genetic variability for shattering resistance present in soybean to support its genetic improvement for resistance to pod shattering. Among the early maturing breeding lines (T1 trial), the breeding TGx2002‐8FM was the most resistant to pod shattering, with shattering scores lower than that of the resistant check Dina. For the medium maturity category (T2 trial), TGx2002‐9FM was the most resistant, with similar resistance to that of Dina. TGx2002‐8FM and TGx2002‐9FM represent valuable germplasm for genetic enhancement of pod shattering resistance in specific maturity group's soybean.

Narrow sense heritability estimates for pod shattering in T1, T2 and T3 trials were 0.80, 0.27 and 0.49, respectively. These estimates were low to high, and consistent with previous estimates (Mohammed et al., 2014; Tukamuhabwa et al., 2000). The wide range of heritability estimates and the significant GE interaction effects on pod shattering observed in this study underscores the sensitivity of pod shattering to environmental conditions and the challenges that these sensitivities may have on effective phenotyping for pod shattering resistance. These challenges can be circumvented through the use of molecular markers linked to major‐effect QTL for pod shattering resistance.

Of the 59 genotypes genotyped, 57 had the *pdh1* resistance allele, while only two lines had the susceptible *Pdh1* allele. A previous study with older IITA germplasm found a higher incidence (~23%) of the susceptible *Pdh1* allele (Miranda et al., 2019). Our results showed a high frequency for the resistance allele in the IITA germplasm, which suggests high selection pressure for this allele. Artificial selection by breeders could have resulted in a selection sweep and near‐fixation for the resistance allelic variant *pdh1*. Given the high priority that is placed on breeding for shattering resistance, and the long period that this selection has been going on in the IITA breeding program, it was not surprising that resistance allele has almost reached a fixation point. During selection for shattering resistance, which has been conducted based on pod shattering phenotype directly, it is likely there was also selection for the *pdh1* resistance allele. The high frequency (near‐fixation of *pdh1*) observed in the current study is consistent with that of Funatsuki et al. (2014) who reported a high frequency of *pdh1* among the old and modern North American germplasm. However, in that same study, they observed a much lower frequency (<20%) among the old and modern Japanese varieties (Funatsuki et al., 2014).

Despite the high frequency of *pdh1* among the 59 genotypes evaluated, a few genotypes lacked this allele, and these genotypes can benefit from introgression of *pdh1* in their background to make them more resistant to pod shattering.

The average pod shattering score for genotypes with the resistance allele *pdh1* was 1.6 compared to 3.1 for genotypes that had the susceptible allele. The t‐test showed that these two average scores were significantly (*p* < .05) different indicating that *pdh1* marker effectively predicted the pod shattering status of the germplasm. This result demonstrated the potential usefulness of *pdh1* marker in MAS for pod shattering to circumvent the challenges associated with phenotypic selection.

Other genetic sources of resistance to pod shattering were not evaluated in this study. There was variation for pod shatter resistance among the germplasm fixed for the *pdh1* gene. In the future, it will be important to determine if the *NST1A* gene for pod shattering resistance or some other uncharacterized genes are controlling pod shattering resistance in the *pdh1* genetic background (Škrabišová et al., 2022).

Breeding for enhanced pod shattering resistance is feasible given the adequate genetic variation that exists for this important trait. However, phenotyping for pod shattering remains challenging because it is time‐consuming, labor‐intensive, and affected by environmental conditions such as ambient temperature and humidity. The marker for pdh1 resistance that has been shown in this study to have high prediction accuracy for pod shattering resistance could play an important role in marker‐assisted selection for pod shattering to circumvent the challenges associated with direct phenotyping. Marker‐assisted selection could also accelerate the development of varieties resistant to pod shattering. Because phenotypic selection for pod shattering can only be done at harvesting maturity, selection at early growth stages using the marker such as the one reported in this study helps to optimize the use of breeding resources such as greenhouse space and time (Kim et al., 2020).

## 5 CONCLUSIONS

There was significant genetic variability for pod shattering resistance among the IITA germplasm. TGx2002‐8FM and TGx2002‐9FM were the most resistant among early and medium maturity genotypes and can be used for genetic enhancement of pod shattering resistance in these specific maturity classes of soybean. Narrow sense heritability estimates for pod shattering ranged from low (0.27) to high (0.80). The resistance allele *pdh1* is highly frequent among the IITA genotypes to the point of reaching fixation, and the marker for this allele effectively predicted resistance to pod shattering. The *pdh1* marker, therefore, can be used in MAS for pod shattering to circumvent challenges of low heritability and significant GE interaction effects associated with direct phenotypic selection for pod shattering.

## CONFLICT OF INTEREST STATEMENT

The authors declare that there is no conflict of interest.

## Data Availability

The data that support the findings of this study are freely available upon request from the corresponding author.
